# Preventive Adolescent Health Care in Family Practice: A Program Summary

**DOI:** 10.1100/tsw.2006.116

**Published:** 2006-06-07

**Authors:** Barry Knishkowy, Moshe Schein, Alexander Kiderman, Aliza Velber, Richard Edman, John Yaphe

**Affiliations:** Family Practice Unit, The Braun School of Public Health and Community Medicine, Hadassah Medical Organization and The Hebrew University, Clalit Health Services and Department of Mother, Child and Adolescent Health, Ministry of Health, Jerusalem, Israel

**Keywords:** adolescents, community oriented primary care, family practice, Israel, preventive care

## Abstract

The AMA Guidelines for Adolescent Preventive Services (GAPS) has been the cornerstone of preventive care for teenagers since its publication in 1994. Despite this, there has been little documentation of their implementation in the family medicine literature. This article gives an overview of a family practice–based adolescent preventive health program based on GAPS recommendations, and reports on compliance, feasibility and health issues. A Community-Oriented Primary Care (COPC) program targeted all adolescent patients aged 12—18 years in two Israeli family practices. 321 teenagers were invited to participate. Every 7th and 10th grader was invited for a preventive health visit with the family physician and nurse. The visits included a medical evaluation, screening and counseling regarding health issues recommended by GAPS, and counseling regarding personal health concerns. Parents were also invited to meet with the staff. 184 (57%) of the adolescents invited for health visits attended. The overall visit time was 47 minutes, including 12 minutes for a questionnaire and 35 minutes with providers. Common biomedical problems included overweight, acne and dysmenorrhea. Health risk behaviors and psychosocial problems included cigarette or alcohol use, dieting, infrequent/never seat belt use, and feeling depressed. 78% wanted to discuss at least one personal health issue. 27% were invited for follow-up visits. Only 3% of the parents came for visits. A community-oriented approach facilitates bringing adolescents for preventive health visits. Many previously undetected health issues, particularly psychosocial and behavioral, are revealed during these visits. A concerns checklist aids in addressing personal health concerns.

## INTRODUCTION

The American Medical Association Guidelines for Adolescent Preventive Services (GAPS)[1] has been the cornerstone of preventive care for teenagers since its publication in 1994. The GAPS

recommendations place high priority on interventions targeting the developmental and behavioral risks that are central to adolescent health. Despite the widespread use of these recommendations, questions remain regarding their feasibility, acceptability, and measurable outcomes[2]. There has been minimal documentation of their implementation in the family medicine literature, and little has been written about confidentiality and other issues that are of particular importance for family physicians providing care to both teenagers and their parents.

Against this background, we developed and implemented the first adolescent preventive health program in an Israeli primary health care setting. Over a period of 5 years, the family physicians and nurses involved in this program provided care to a defined population of teenagers in two Israeli clinics. In this article, we give an overview of the program's development and structure, the major findings, and the implications for adolescent health care in family practice.

## METHODS

The Hadassah Community Health Center, located in Western Jerusalem, has been a major site for the development and teaching of Community Oriented Primary Care (COPC) since the 1960s. The COPC approach identifies and prioritizes the major health issues of an entire population group under the health team's care, and allows for the development and evaluation of programs targeting these issues[3,4,5].

A study of adolescent visits to the Center's family practice clinic during the 1980s showed that teenagers visited relatively infrequently, coming mainly for acute, biomedical problems[6]. For these reasons, the structured framework and the outreach implicit in the COPC approach were particularly relevant to the adolescent population.

In order to establish a "case for action" for a preventive adolescent health program, focus groups and individual discussions were held with the family physicians and nurses, teenage patients, parents, and a boarding school's staff and students who received their care in the family practice clinic. The discussions focused on perceptions of the major health issues of adolescents, overall interest in establishing a preventive service, and opinions related to confidentiality and sharing information[7].

A physician and nurse coordinator worked together to develop protocols, coordinate the staff work, and monitor the program. The Family Medicine Unit's staff met several times to discuss program objectives and methodology. They received ongoing training in adolescent medicine issues and specific instruction regarding program protocols.

The program was introduced in three phases. In the first phase, beginning in 1993, the boarding school teenagers began to participate in the program[7]. The second phase, beginning in 1995, added the adolescent patients who lived in the community with their parents ("neighborhood teenagers"). The third phase, beginning in 1997, entailed addition of the adolescent population of one family physician working in a more typical clinic setting. This allowed for testing the program in the milieu in which most Israeli family physicians practice.

### Population

The Hadassah Community Health Center Family Practice (FPC-1) population included approximately 650 teenagers aged 12–18 years; 300 were pupils at the boarding school and 350 lived with their parents in the neighborhood. The second family practice (FPC-2) was located approximately 10 km from Jerusalem. The target population included approximately 150 adolescent patients. Both clinics were located in lower middle class, Jewish neighborhoods. All patients had health insurance coverage and all clinic visits were free of charge.

## Staff

The family practice staff at FPC-1 included four physician-nurse teams, each of which was responsible for the care of a defined population. The family physician in FPC-2 worked individually, as in the typical Israeli family practice setting.

## Overall Program Structure

All adolescents listed as patients in the family practice population were invited for a comprehensive health visit with the family nurse and family physician when they were in the 7th and 10th grades. The visits were conducted in the health center after school hours during specially allotted times. The program and study were approved by the Research Ethics Committee of the Hadassah Hospital.

## Invitation Process

According to the program protocol, an invitation letter, including a consent form to be signed by the parents, was sent to each teenager from the "neighborhood" population. Appointments were scheduled for those teenagers who responded or whose parents responded for them. After a minimum of 3 weeks, the family nurse (or physician, in the case of Clinic B) called every teenager who had not responded to set up an appointment. Parents were sent a separate letter that included a letter of explanation, a checklist of topics that they would like to be discussed with their child, and an invitation for the parents to meet with the physician or nurse. During the first year that "neighborhood" teenagers were invited, this letter and the consent form were sent before any contact was made with the teenagers themselves. Only after receiving parental consent was the teenager called to set up an appointment[8].

## Content

The health topics selected for screening and counseling were similar to those recommended in GAPS and included growth and development issues, injury prevention, nutrition and physical activity, dental health, smoking, alcohol and substance abuse, depression, cardiovascular risk factors, and sexual behavior (10th grade only). Counseling regarding individual health concerns was also a major focus of the visits.

## Intake

The health visit began with the adolescent's completing a self-administered questionnaire that inquired about personal and family health issues and health behaviors. The questionnaire also included a checklist of 92 topics that may "concern" adolescents or about which the teenager may want to receive information[9]. After circling the topics of personal concern, the adolescents were asked to list up to three topics from the list that they would like to discuss with the family physician or nurse during the visit. Finally, the younger adolescents were asked to complete a pubertal self-assessment using pictures of sexual maturity ratings according to Tanner[10]. The questionnaire was followed by a structured interview by the nurse (or physician, in the case of Clinic B). This included a general medical history, an adaptation of the HEADSS psychosocial interview[11], and counseling regarding the target health issues, according to a defined protocol. The nurse or physician also addressed the topics that the individual teenager had requested for discussion. The family physician completed the visit with a review of any problems that arose during the questionnaire or nurse interview, a physical examination (including genital examination for the boys), discussion of any remaining topics, and plans for any follow-up that was required.

## RESULTS

Throughout the 5 years (1993–1998) of the program, 321 teenagers were invited to participate, and 184 of these (57%) attended. Attendance rates according to clinic group, gender, and grade are presented in Table 1. Most of the participants (81%) were 7th graders, reflecting the fact that all the boarding school students who attended were invited during their first year (7th grade) at the school. Nearly all of these students came for the visits, as they were expected to attend and were brought to the clinic by their staff members.

**TABLE 1**
**Study Population**

| Variable | Number Invited (%) | Number Attended (%) | % Attended |
|---|---|---|---|
| **Clinic group** | | | |
| FP1 – boarding school | 114 (35.5) | 96 (52.2) | 84.2 |
| FP1 – neighborhood | 155 (48.3) | 63 (34.2) | 40.6 |
| FP2 | 52 (16.2) | 25 (13.6) | 48.2 |
| **Sex** | | | |
| Male | 168 (52.3) | 94 (51.1) | 56.0 |
| Female | 153 (47.7) | 90 (48.9) | 58.8 |
| **Grade** | | | |
| 7th | 225 (70.1) | 149 (81.0) | 66.2 |
| 10th | 96 (29.9) | 35 (19.0) | 36.5 |
| **Total** | 321 (100.0) | 184 (100.0) | 57.3 |

Among the "neighborhood" teenagers, no differences in compliance were found according to gender, grade, or any of the other demographic variables that were studied. There were differences in the attendance rates among the five health care teams, and lower attendance rates among adolescents with unknown (vs. high or normal) risk status, as determined by the nurse or physician before the invitation process[12]. Following the written invitation with a phone call more than doubled the percentage of neighborhood patients who attended the visits[8].

The time required for the health visits was recorded during the first 53 encounters. The overall visit time was 47 min, with 12 min required for the self-administered questionnaire, 21 for the nurse interview, and 14 for the physician examination[7].

The principal biomedical issues revealed during the health visits were overweight (BMI > 85%, 11% of the boys and 18% of the girls), acne (28% of the boys and 33% of the girls), and dysmenorrhea (31% of the girls). Approximately half of those with acne or moderate to severe dysmenorrhea had never been treated. Less common diagnoses made during the physical examination included heart murmur (12 considered to be functional, and 1 referred for further evaluation and found to be functional), scoliosis (10 considered to be insignificant and 1 referred to an orthopedist), and elevated blood pressure (2 cases).

Selected health behaviors, according to grade and gender, are displayed in Table 2. Only two reported to having tried illegal drugs. However, alcohol consumption was extremely prevalent among these teenagers, 23% reported feeling depressed in the past month, and 4 had attempted suicide.

**TABLE 2**
**Selected Health Risk Behaviors (Percentages)**

| Health Risk Behavior | 7th Grade Boys | 7th Grade Girls | 10th Grade Boys | 10th Grade Girls |
|---|---|---|---|---|
| Ever dieted | 13.9 | 43.2 | 22.2 | 62.5 |
| No dental visit – past 2 years | 15.1 | 17.8 | 27.8 | 12.5 |
| Ever smoked cigarette | 11.1 | 4.2 | 61.1 | 50.0 |
| Drinks alcohol | 29.7 | 16.9 | 77.8 | 50.0 |
| Front seatbelt – infrequently or never* | 9.5 | 4.3 | 5.6 | 6.3 |
| Rear seatbelt – infrequently or never* | 40.9 | 43.5 | 77.8 | 56.3 |
| Had sexual intercourse | - | - | 20.0 | 22.2 |
| N | 75 | 74 | 19 | 16 |

*Based on a consecutive sample of 79 visits.

Many (78%) of the teenagers wanted to discuss some health issue with the family physician or nurse during the health visit. Of these, 14% requested one topic, 16% requested two topics, and 70% requested three topics. There was a wide range of topics that interested them. Fig. 1 presents the categories of issues requested for discussion by the 7th graders, according to gender.

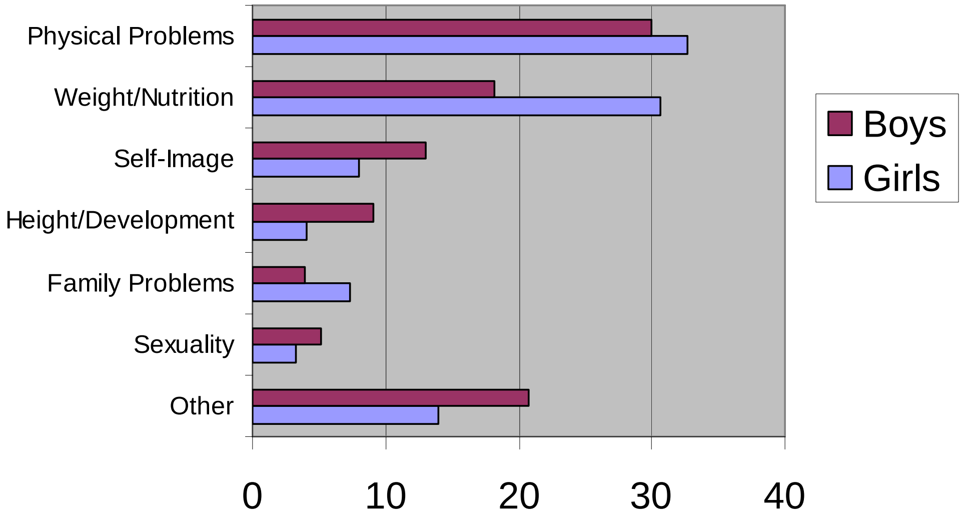

**FIGURE 1**. Topics requested for discussion; 7th graders (n = 149).

A total of 50 participants (27%) were invited for follow-up visits. Of these, 30 were invited to return to the nurse, 18 to the physician, and 2 to both the nurse and physician. Of the teenagers invited back, 20 had more than one reason for returning, giving a total of 91 diagnoses requiring follow-up. The principal reasons for follow-up visits are presented in Table 3.

No differences in compliance measures were found using two different invitation protocols, where either parents or teenagers were contacted initially about the program[8]. Thirty-seven (18%) of the parents returned questionnaires regarding topics that they wanted to be discussed with their children. Only 6 (3%) came for visits with the family practice staff..

**TABLE 3**
**Reasons for Follow-Up Visits**

| Diagnosis | Number of Teenagers n (%) |
|---|---|
| Acne | 12 (13.2) |
| Overweight | 8 (8.8) |
| Depression | 8 (8.8) |
| Dysmenorrhea | 8 (8.8) |
| Alcohol use | 6 (6.6) |
| Cardiovascular risk | 6 (6.6) |
| Unhealthy dieting | 5 (5.5) |
| Other | 38 (41.8) |
| Total number of diagnoses | 91 (100.0) |
| Total number of teenagers invited | 50 (27.2) |

n = 184.

## DISCUSSION

In this paper, we have described the development and implementation of a COPC program for adolescents in the primary care setting. Adolescents visit physicians less frequently than other age groups[13,14] and the reasons for these visits are characteristically for biomedical issues. Many are hesitant to discuss health risk behaviors with clinicians[15]. The COPC approach, which involves outreach to the entire population and the prioritization of health interventions, is therefore particularly relevant to this age group.

Addressing and treating certain physical problems such as overweight, untreated dysmenorrhea, and untreated acne, proved to be important for many teenagers who had previously avoided or not gotten around to seeking medical attention. However, major pathological conditions were not detected in this population. On the other hand, psychosocial and behavioral problems such as lack of seatbelt use, unhealthy dieting, smoking and alcohol use, were frequent issues requiring intervention. Assessing and counseling regarding family problems and depression were also quite common, and the focus of many visits.

Using a concerns checklist was an invaluable tool that turned the visits into “patient-oriented” encounters. This tool, which is part of the initial intake questionnaire in many adolescent clinics, is often used to screen for biomedical or psychosocial problems[9,16]. We have used it for an additional purpose — showing the teenager that the health team is ready to discuss a wide range of issues and allowing them to make their personal concerns the focus of the visit. In fact, data from a U.S. national sample of in-school adolescent boys and girls found that the topics most frequently discussed with their providers were not necessarily those that they were most interested in discussing[17]. Our staff’s impression was that this was the most important part of the program for many of the participants.

Several studies of U.S. physicians have looked at compliance with the GAPS recommendations. These surveys have shown screening and counseling rates that range from less than 20% to over 60% for specific health issues[13,18,19,20]. One study of family physicians showed that health-habit counseling was provided during only 38% of the visits[21]. It should be emphasized that these inadequate rates relate to adolescents who already visit their physicians and not to the entire, possibly at higher risk, practice population.

Most Israeli teenagers, including those in the study population, do not visit their physicians routinely for preventive health visits[6]. In our community-oriented program, targeting all teenagers registered as patients, the compliance rate for the “neighborhood” population was nearly 50%. We consider this to be a relative success in introducing preventive care to a population unaccustomed to the concept. Invitation strategies other than that used in this program, such as personal invitations to return for preventive visits when adolescents come for acute care and contacting the remaining adolescent patients by mail or phone, may result in similar or better compliance rates. Additional efforts are probably necessary to ensure that teenagers at highest risk are among those brought into the program.

Time demands are one of the factors influencing compliance rates. One previous study has shown that the addition of counseling to any extent increased the average length of a physician visit from 13.8 to 17.6 min[13]. In our program, the average time for a preventive visit was 47 min, with 35 min spent with the nurse or physician. In addition, 27% of the participants required follow-up visits. This time investment, while in part explained by the academic aspects of the program, is not possible in most primary care settings. We believe that the GAPS recommendations, which include comprehensive, annual counseling, should be limited in order to be a feasible component of adolescent primary health care. Our more recent experience with a modified program has shown that 20–25 min are adequate for most preventive visits.

Many physicians in Israel[22] and elsewhere are inadequately trained in adolescent medicine. Moreover, provider-perceived self-efficacy to deliver adolescent preventive services is related to actual screening rates[23]. Physician training can improve the situation; in fact, continuing education sessions on preventive care have been shown to increase the delivery of adolescent preventive health services[24].

There were several limitations in the implementation and evaluation of this program. First, the population was overly represented by 7th graders, mainly due to the fact that the boarding school did not send their 10th graders for the preventive visits. This in no way diminishes the importance of preventive visits during the high school years. Second, we consider parental participation to be of great importance and need to develop strategies to ensure a higher level of parental involement. Finally, we were not able to systematically evaluate the effectiveness of the program's interventions. Data of this sort are sparse, and collecting them constitutes a major challenge for adolescent primary care health providers.

Table 4 highlights several of the lessons learned about implementing a program for adolescent patients in family practice. Other factors that can enhance a program's success include creating an office environment receptive to adolescents, developing clinic policy that permits adolescents to receive confidential health care, and selecting a preventive services coordinator[25]. In Israel specifically, financial incentives for physicians performing preventive visits, and limiting the scope and time of the physician visits (nurse-physician teams are the exception to the rule in Israeli clinics), would increase the feasibility of this type of program.

**TABLE 4**
**Lessons Learned from the Program**

- Clinicians, parents, and adolescents should be involved in program development.
- A community-oriented approach may help to reach the teenagers at highest risk. Different invitation strategies should be tried in order to maximize attendance rates.
- Physician-nurse teamwork promotes efficiency and quality of care.
- Psychosocial and behavioral issues are common. They may be screened for with a health questionnaire and a psychosocial interview.
- Patient-oriented care is facilitated using a "concerns checklist".
- GAPS recommendations may need to be limited in order to be feasible.
- Preventive adolescent health visits reveal previously unknown problems and concerns. They provide a valuable service to many teenagers.

Adolescent preventive health services in the primary care setting have a great potential for reducing adolescent morbidity and for addressing the health concerns of teenagers. We have summarized a family practice program that targeted an entire population of adolescents and attempted to provide comprehensive preventive services within the context of invited health visits. The accomplishments and difficulties that we have described may be instructive for family physicians in designing similar, essential services for their teenage patients.

## ACKNOWLEDGMENTS

The authors gratefully acknowledge the contributions of professor Hava Palti in guiding the program development; family nurses Galit Cnaan, Pnina Naveh, Miri Haringman, Hana Gordon; Dr. Vered Oppenheimer-Gazit in development of the program's monitoring system; Dr. Mario Baras and Rachel Goldwag for statistical support; and the entire staffs of the Hadassah Community Health Center's Family Practice Unit and the Adolescent Health Center of the Hadassah Social Medicine Department and the Jerusalem Municipality for their support and consultations.

## BIOSKETCHES

**Barry Knishkowy**, **MD, MPH**, Adolescent Health Coordinator in the Department of Mother, Child and Adolescent Health at the Israel Ministry of Health and a family physician with Terem Family Practice in Jerusalem, Israel. E-mail: barry.k@moh.health.gov.il

**Moshe Schein**, **MBChB, MS,** a family practitioner at the Hadassah Hebrew University Medical Center in Jerusalem, Israel. Research interests include the treatment of hypertension and care of the elderly. E-mail: mhschein@yahoo.com

**Alexander Kiderman**, **MD,** a family physician with the Division of Family Medicine, Hebrew University, Hadassah Medical School and Clalit Health Services, Jerusalem, Israel. E-mail: kider@cc.huji.ac.il

**Aliza Velber**, **RN,** a retired family nurse, from the Family Medicine Unit at the Hadassah Community Health Center, Jerusalem, Israel. E-mail: avramv@013.net

**Richard Edman**, **MD**, a family physician with the Maccabi Health Services in Haifa, Israel. Research interests include early cancer detection programs and melanoma prevention. E-mail: edman2@012.net.il

**John Yaphe**, **MD, MClSc**, Director of Research in the Department of Family Medicine, Rabin Medical Centre, Petach Tiqva, Israel. E-mail: yonay@clalit.org.il
